# The Resignations at the Sheffield Union Hospital

**Published:** 1907-04-27

**Authors:** 


### THE RESIGNATIONS AT THE SHEFFIELD UNION HOSPITAL.

We have lately received a cutting from the *Sheffield Independent* of April 13, in which attention is drawn to a serious state of affairs now existing at the Sheffield Union Hospital. According to the writer of the article, the three medical officers attached to this institution have recently resigned. This, in itself, would merely point to some grave dispute between the residents and the hospital committee, for which either party might be to blame. But when the article goes on to say that no fewer than eight medical residents have left this hospital since June 29 last, it becomes clear that there must be something fundamentally wrong with the attitude of the hospital authorities towards the resident medical staff.

It is almost inconceivable that in nine months eight medical men should resign important offices in the same hospital without adequate reasons for so doing. We cannot, in justice to our profession, assume that the fault lies anywhere but at the doors of the Sheffield Union Hospital Committee. Wounded vanity or petty resentment might account for one, or even two, of these sudden exits —such things have happened before in other hospitals, and will no doubt happen again—but it would be both wrong and foolish to apply this explanation to a series of eight resignations.

Inquiries made by the representative of the *Sheffield Independent*, among those behind the scenes, have brought to light certain facts which, in the absence of evidence to the contrary, support our belief that the hospital committee are entirely to blame for the present *fiasco*. It appears that in March 1906 the Local Government Board issued special orders, intended to increase the authority and strengthen the position of the medical officers of this institution. These decrees found so little favour in the eyes of the committee that it only published that part which defined the actual duties of the doctors, and suppressed the sections showing that the medical staff had the supervision of other officials. At every point the committee seem to have disobeyed the spirit of the Local Government Board's commands.

The article then enumerates several incidents, which, if they are true, are sufficient in themselves to justify the resignation of successive medical officers. The committee really seem to have neglected no detail which could possibly cause friction between the medical and administrative staffs, and to have done everything in their power to weaken the authority and ignore the rights of the resident medical officers. And, as always happens in such cases, the real sufferers have been the 400 patients, who, at the time this article was written, were presumably left without any organised medical attention whatsoever. For their sake it is to be hoped that the local practitioners were willing to give temporary aid to them in their unfortunate plight. But we trust that the committee have been less successful in finding candidates for the posts of resident medical officers, in the absence of an express undertaking on their part to amend the error of their ways.

